# Dietary tomato inhibits angiogenesis in TRAMP prostate cancer but is not protective with a Western-style diet in this pilot study

**DOI:** 10.1038/s41598-021-97539-2

**Published:** 2021-09-17

**Authors:** Catherine C. Applegate, Matthew R. Lowerison, Emma Hambley, Pengfei Song, Matthew A. Wallig, John W. Erdman

**Affiliations:** 1 Division of Nutritional Sciences, University of Illinois at Urbana-Champaign, Urbana, IL 61801 USA; 2 Beckman Institute for Advanced Science and Technology, University of Illinois at Urbana-Champaign, Urbana, IL 61801 USA; 3 Department of Electrical and Computer Engineering, University of Illinois at Urbana-Champaign, Urbana, IL 61801 USA; 4 Cancer Center at Illinois, University of Illinois at Urbana-Champaign, Urbana, IL 61801 USA; 5 Department of Pathobiology, College of Veterinary Medicine, University of Illinois at Urbana-Champaign, Urbana, IL 61801 USA; 6 Department of Food Science and Human Nutrition, University of Illinois at Urbana-Champaign, Urbana, IL 61801 USA; 7 Present Address: Division of Biology, Kansas State University, Manhattan, KS 66506 USA

**Keywords:** Tumour angiogenesis, Urological cancer, Cancer, Cancer imaging

## Abstract

Prostate cancer (PCa) remains the second most diagnosed cancer worldwide. Higher body weight is associated with chronic inflammation, increased angiogenesis, and treatment-resistant tumor phenotypes. Dietary tomato reduces PCa risk, which may be due to tomato inhibition of angiogenesis and disruption of androgen signaling. This pilot study investigated the interplay between tomato powder (TP), incorporated into control (CON) and obesogenic (OB) diets, and PCa tumor growth and blood perfusion over time in a transgenic model of PCa (TRAMP). Ultrasound microvessel imaging (UMI) results showed good agreement with gold-standard immunohistochemistry quantification of endothelial cell density, indicating that this technique can be applied to non-invasively monitor tumor blood perfusion in vivo. Greater body weight was positively associated with tumor growth. We also found that TP significantly inhibited prostate tumor angiogenesis but that this inhibition differentially affected measured outcomes depending on CON or OB diets. TP led to reduced tumor growth, intratumoral inflammation, and intratumoral androgen-regulated gene expression (*srd5a1*, *srd5a2*) when incorporated with the CON diet but greater tumor growth and intratumoral gene expression when incorporated with the OB diet. Results from this study show that protective benefits from dietary tomato are lost, or may become deleterious, when combined with a Western-style diet.

## Introduction

Despite declining trends in diagnoses, prostate cancer (PCa) remains the second most diagnosed cancer worldwide and fifth leading cause of cancer-related deaths among men^1^. Although highly curable in early stages, advanced stage PCa is associated with a high risk of metastases and resulting dramatic reductions in 5-year survival rates^2^. Smoking, diet, and increased body mass index (BMI) are all modifiable risk factors for advanced stage PCa^3^. Treatment typically involves androgen deprivation therapy (ADT) to pharmaceutically inhibit the production of androgens, such as testosterone, that are important for PCa development and growth. Importantly, individuals with obesity tend to present with lower serum testosterone, which may promote the initial growth of more aggressive, high-grade PCa phenotypes that are less responsive to ADT^4–6^.

Angiogenesis, one hallmark of cancer^7^, is upregulated in prostate tumors at all stages of development. Microvessel density (MVD) is reportedly increased in PCa tumors^8^ and has been associated with advanced stage disease^9^, increased metastases^10^, and shorter survival time^11^. Moreover, increased visceral adipose tissue, such as periprostatic adipose, may increase the risk of PCa recurrence and poorer prognostic outcomes^12^. Adipose secretes pro-inflammatory cytokines, such as tumor necrosis factor-α (TNFα), that stimulate cell proliferation and angiogenesis in PCa lesions^13–15^. Additionally, adipose tissue expansion leads to hypoxia, promoting adipocyte crosstalk with vascular stromal cells to stimulate additional angiogenesis, which in turn promotes tumor growth^16^.

Despite this evidence and the limited efficacy of some clinical trials testing anti-angiogenic therapies in PCa^17,18^, the majority of phase III trials do not support improvements in overall survival by inhibiting angiogenesis^19–22^. Epidemiological studies have demonstrated that intake of tomato and its primary bioactive, lycopene, reduce the risk for PCa-specific mortality^23,24^. Higher dietary intake of lycopene is associated with reduced angiogenesis in PCa tumors^23^; this epidemiological relationship is supported by mechanistic studies in which lycopene treatments inhibited angiogenesis and angiogenic factors in vitro and in vivo^25–27^. Furthermore, tomato and lycopene appear to interact with the androgen axis in PCa, disrupting androgen metabolism through downregulation of the androgen receptor (AR) and AR-regulated steroid-metabolizing enzymes^28^.

Although the monitoring of prostate tumor blood perfusion is not a current standard of care, contrast-enhanced ultrasound imaging studies support an association between greater MVD and increased Gleason score at the time of tumor biopsy^8^. Hemodynamic indices measured in prostates also revealed lower vascular perfusion in non-malignant tissues and low-grade tumors compared with malignant tissues and high-grade tumors, respectively^29^. While ultrasound imaging is a convenient and affordable tool to monitor prostate tumor growth, the use of contrast agents requires invasive techniques and additional specialized personnel. However, we recently published a description of a non-invasive super-fast ultrasound microvessel imaging (UMI) technique that can detect slow flowing blood through small vessels^30^. UMI can recreate an image depicting the vasculature network of a tissue without the use of contrast microbubbles and therefore has vast potential for monitoring angiogenesis in vivo. This technique was implemented in this study using a commercially available high-frequency ultrasound system (FUJIFILM VisualSonics Vevo 2100) that is capable of exporting pre-envelope-compressed in-phase/quadrature (IQ) datasets.

The objectives of this pilot study were to validate the use of the UMI technique for detecting longitudinal changes in blood perfusion of autochthonously-developed PCa (transgenic adenocarcinoma of the mouse prostate; TRAMP model), as well as to determine the effects of diet-induced obesity and tomato intake on PCa growth and tumor blood perfusion. The TRAMP model was selected as it mimics the pathogenesis of human PCa, which is critical in determining clinically translatable associations with dietary intake. TRAMP mice bred on an FVB background tend to develop well-differentiated adenocarcinomas that progress to poorly differentiated adenocarcinomas with a neuroendocrine (NE) phenotype^31,32^, similar to the progression of advanced or treatment-resistant PCa observed in humans^33,34^.

Dietary tomato, when incorporated into either control or obesogenic diets, resulted in significantly reduced tumor blood perfusion over time as measured by UMI and a reduction in VEGF expression. While we expected to observe protective effects by dietary tomato across both control and obesogenic diets, tomato paradoxically led to reduced tumor volume when incorporated into a control diet yet greater tumor volume when incorporated into an obesogenic diet. Similar molecular trends were observed in which tomato inhibited markers of inflammation, hypoxia, and androgen metabolism when combined with a control diet but increased these markers when combined with an obesogenic diet. UMI was consistent with histological features of angiogenesis, indicating that contrast-free UMI can be used in conjunction with ultrasonic tumor volume monitoring to non-invasively detect small changes in tumor blood perfusion and tumor volume over time in response to anti-angiogenic therapies. Results from this study additionally show that protective benefits from dietary tomato are lost, or may even become deleterious, when combined with a Western-style diet.

## Methods

### Diets and carotenoid measurement

Diets were either control (CON) or obesogenic (OB) both with and without 10% tomato powder (TP). A level of 10% TP in mice translates to 17 mg of lycopene per day (0.24 mg/kg) for a 71-kg man^35^, which equates to a ½ cup serving (123 g) of tomato sauce per day. CON diets were modified AIN-93G diets (17.2% kcal from fat), and OB diets were selected to mimic a high fat/sugar Western-style diet consumed by humans (Teklad DIO #08811; 44.6% kcal from fat). Diets were powdered to incorporate 10% lyophilized tomato paste (Port Royal Premium California Fancy Tomato Paste, Woodbury, NY, USA) and adjusted to ensure consistent macronutrient distribution within each diet type (CON or OB, Supplementary Table S1). Samples from each prepared batch of diet stored at -20ºC until analyzed for carotenoid content via high-performance liquid chromatography (HPLC) using previously described methods^36–38^. Analyzed carotenoids included all-*trans* lycopene, total *cis* isomers of lycopene, phytoene, phytofluene, α-carotene, β-carotene, β-cryptoxanthin, lutein, and zeaxanthin. Diet extracts were injected onto an Alliance HPLC system (e2695 Separation Module) equipped with a 2998 photodiode array detector (Waters, Fisher-Scientific) within 24 h of extraction. Extracts were separated on a reverse-phase C30 column (4.6 × 150 mm, 3 μm; YMC, Kyoto, Japan) maintained at 18 °C using a phase gradient method for carotenoid separation described by Yeum et al. (1996)^39^.

### Animals

The University of Illinois Laboratory Institutional Animal Care and Use Committee reviewed and approved all animal procedures (protocol #19011), which were carried out in accordance with the National Institutes of Health Guide for the Care and Use of Laboratory Animals and reported according to the ARRIVE guidelines. Male C57Bl/6-Tg(TRAMP)8247Ng/J and female FVB/NJ mice were purchased from The Jackson Laboratory (Bar Harbor, ME, USA) and bred to produce offspring ([C57Bl/6 × FVB]F_1_). The hemizygous TRAMP genotype was confirmed by tail DNA analysis, which was isolated with Extract-N-Amp Tissue PCR kits (Sigma-Aldrich, St. Louis, MO, USA) and genotyped according to the primer sequences listed in Supplementary Table S2. Mice were housed under controlled conditions (12-h light/dark cycle, 22ºC, 55% humidity), weighed weekly, and given fresh diet three times per week.

### Randomization and euthanasia

**Figure 1:**
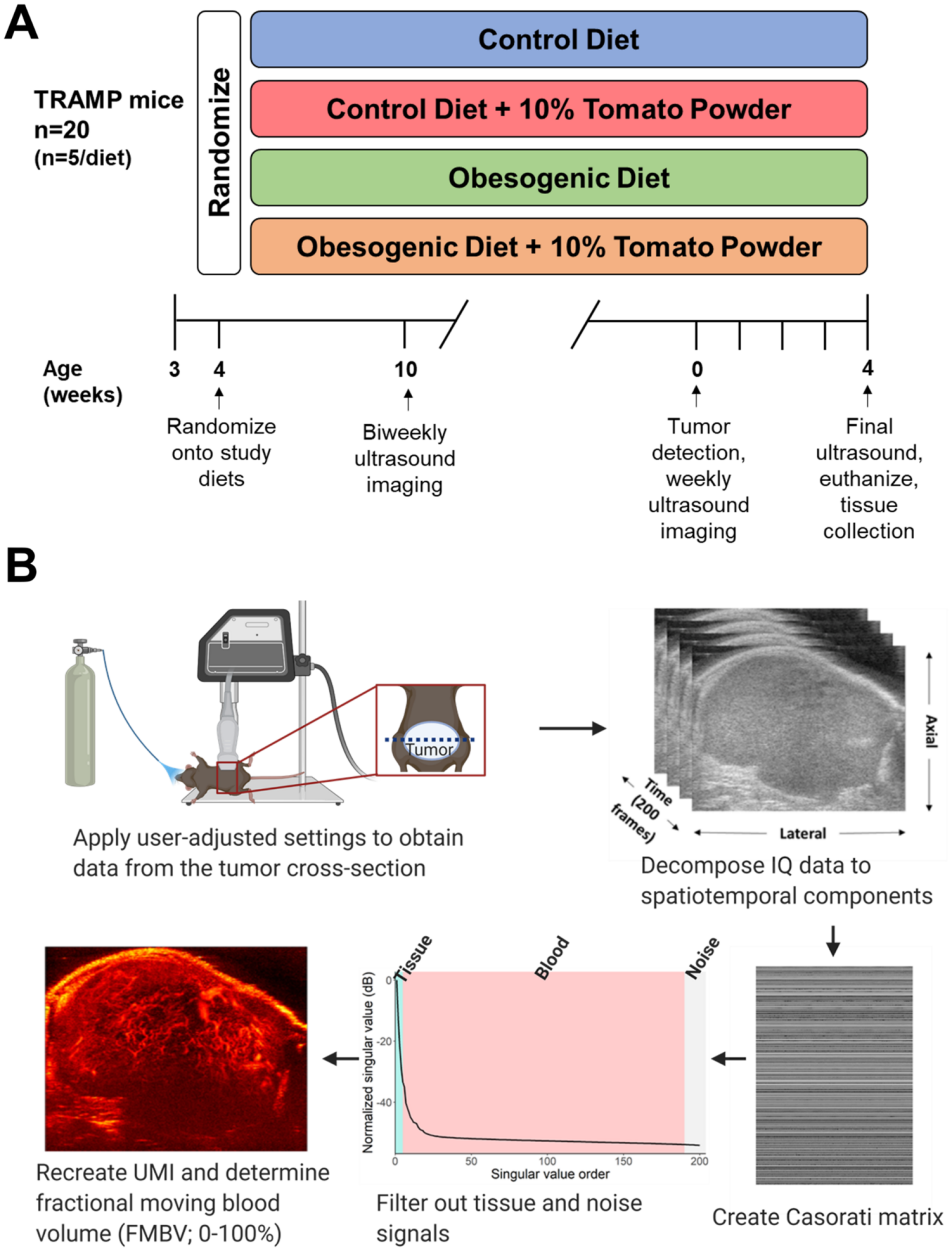
(**A**) Study design. (**B**) Summary of ultrasound microvessel imaging (UMI) technique.

Mice were weaned at 3 weeks of age and acclimated to a powdered AIN-93G diet for 1 week, after which time mice were randomly assigned (by random number generator) to consume one of four diets (n = 5/diet): CON, CON + 10% TP (CON-TP), OB, and OB + 10% TP (OB-TP) (Fig. 1A). Following ultrasonic tumor detection, mice were monitored weekly for 4 additional weeks (for a total of 5 weeks of tumor growth). Euthanasia occurred under anesthesia (isoflurane) by cardiac puncture and cervical dislocation. Tumor tissues were collected, immediately snap-frozen in liquid nitrogen, and stored at − 80 °C until analysis. A center section of each tumor was also immediately fixed in formalin for histological processing.

### Ultrasound imaging

Tumor development was monitored via bi-weekly (every other week) ultrasound imaging (FUJIFILM VisualSonics Vevo 2100, Toronto, Canada) starting at 10 weeks of age and until tumor detection, at which time ultrasound imaging was performed weekly for 4 additional weeks. Mice were anesthetized and maintained under 2% isoflurane (or to effect) and 1–2% O_2_. Depth of anesthesia was monitored by pedal reflex and respiratory rate. Hair over the lower abdomen was removed by a depilatory agent, and ultrasonic scans were obtained through the ventral side while animals laid in dorsal recumbency on a heated table. Scans were conducted using a 40 MHz linear array transducer (MS-550-D, VisualSonics) in 3D B-mode, and frames were collected in a caudal to cranial direction at intervals of approximately 0.152 mm as previously described^40^. The prostate was identified by using the urethra as an initial landmark, with the endpoint of the volume being the anechoic bladder. Tumors presented as stiff hypoechoic spherical masses in contrast to the surrounding prostate tissues. Serial 2D image slices were then used to generate tumor volume estimates (mm^3^) as previously described^41^.

After each volumetric tumor scan, additional IQ-data ultrasound cineloops were acquired using the VisualSonics “RF-capture mode” for microvessel image processing. For IQ-data capture, the transducer was positioned at the largest, center cross-section of the tumor, and operator settings were adjusted to maximize frame rate by reducing the number of transmit focal zones to one. Interference by animal movement was minimized by truncating the acquisition to 100 frames that excluded respiration motion. IQ-data was exported using the VisualSonics VevoLAB software for off-line processing with customized software in MATLAB (v. 9.6, MathWorks, Natick, MA, USA). Tumor ultrasound microvessel imaging (UMI) was performed as previously described^30^ for each ultrasound cineloop at each timepoint and summarized in Fig. 1B. Briefly, IQ data for each image was reshaped into a Casorati matrix and then decomposed into spatiotemporal components using singular value decomposition (SVD) filtering. SVD clutter filtering thresholds were applied to suppress tissue (the first 5 singular values) and noise signals (the last 10 singular values), and an inverse SVD was performed to isolate spatiotemporal blood flow signals. The filtered Casorati matrix was reshaped to the original IQ-data dimensions and summed along the temporal dimension to result in a microvasculature image. A noise equalization profile^42^ was estimated using the last 10 singular values and applied to correct for depth-dependent attenuation. A region of interest (ROI) was manually delineated to segment out the tumor cross-section from the surrounding prostate tissue. Fractional moving blood volume (FMBV) was calculated using the method introduced by Rubin et al.^43^ within the tumor ROI to measure total tumor blood perfusion. FMBV was scaled from 0 to 100% using the highest detected power as a normalization reference standard. The change in FMBV (ΔFMBV) was calculated as the difference in FMBV from tumor detection to final (week 5) tumor scan (FMBV_detection (det)_ – FMBV_final_ = ΔFMBV).

### Molecular analyses

Tumor tissues were fixed in 10% neutral-buffered formalin for 24 h and then transferred to 70% ethanol for short-term storage. Fixed tissues were embedded in paraffin, and 4 µm slices were stained with hematoxylin and eosin (H&E) for histologic evaluation to determine grade and general morphologic features, performed in blinded fashion by a board-certified veterinary pathologist (MAW). Additional slices were stained against and evaluated for tumor macrophage infiltration (anti-F4/80; Cell Signaling, Danvers, MA, USA) and blood vasculature (anti-factor viii; Agilent, Santa Clara, CA, USA). Slides were digitized (Nanozoomer, Hamamatsu, Japan), and immunohistochemical data were quantified for intensity of staining (optical density) and the area stained (MVD) using CellProfiler (v. 3.1.9, Broad Institute, open-source) and ImageJ/Fiji software (v. 1.52, NIH, Bethesda, MD, USA). Blood vessel architecture was evaluated and measured, and regularity of the lumina was calculated as: perimeter^2^/(4 × π × area), with a value of 1.0 indicating a perfect circle and values > 1.0 indicating increasing irregularity.

For protein analysis, tumor tissues (~ 20 mg) were homogenized with RIPA lysis buffer (Thermo Fisher Scientific, Waltham, MA, USA) and protease/phosphatase inhibitor cocktail (Cell Signaling), and the resulting protein-containing supernatant was collected and stored at – 80 °C. Total protein in each sample was measured by BCA Assay (Thermo Fisher Scientific) according to the manufacturer’s instructions. Western blot was used to quantify vascular endothelial growth factor (VEGF; Abcam, Cambridge, MA, USA, 1:250 dilution) and hypoxia-induced carbonic anhydrase 9 (CA9; Abcam, 1:250 dilution) expression relative to the housekeeping protein α-tubulin (Cell Signaling, 1:1000 dilution), and TNFα was quantified by ELISA according to the manufacturer’s instructions (R&D Systems, Inc., Minneapolis, MN, USA).

For gene expression, total RNA was extracted from ~ 30 mg tumor tissue using RNeasy kits (Qiagen, Valencia, CA, USA) according to the manufacturer’s instructions. RT^2^ First-strand kits (Qiagen) were used to synthesize cDNA, and targeted quantitative polymerase chain reaction (qPCR) was carried out with SYBR Green chemistry (Qiagen) in a QuantStudio 7 instrument (Thermo Fisher Scientific) using validated forward and reverse primers (Integrated DNA Technologies, Inc., Coralville, IA, USA) to measure the expression of steroid-regulated genes: 5α-reductase 1 and 2 (*srd5a1*, *srd5a2*) and the *ar* according to primer sequences listed in Supplementary Table S2. Gene expression was normalized to the housekeeping gene *rpl19* and calculated using the 2^−ΔΔCt^ relative expression method.

### Statistical analysis

All statistics were carried out blindly using either SAS (v. 9.4, Cary, NC, USA) or GraphPad Prism (v. 8.3.0, San Diego, CA, USA). Data were analyzed to determine differences between CON diets (without TP + with 10% TP; CON-All) and OB diets (without TP + with 10% TP; OB-All) and to evaluate the impact of 10% TP on outcome measures both independent of TP (No TP vs. TP) as well as within CON and OB diets (interaction effect of diet*TP). Body weight at tumor detection was compared within and between dietary groups using mixed model two-way ANOVA with multiple-comparison adjustments by Sidak’s test. Generalized linear mixed models were created to determine the effect of different diet levels (diet, TP, and diet*TP) on weekly tumor volume (mm^3^) measurements in tumor-bearing mice. Due to the exponential rate of weekly volume increase and the heterogeneity between individual measurements, tumor volumes were log-transformed (log[mm^3^]) for statistical analyses. A random intercept and slope (time) with subject (mouse) and treatment effects were included within the models. To separate the effects of diet and body weight, body weight analyses were adjusted to account for dietary intervention, while age at tumor detection, tumor growth measurements, tumor weight at euthanasia, FMBV measurements (FMBV_det_, FMBV_final_, ΔFMBV), immunohistochemistry, protein, and gene expression data were adjusted to include body weight at tumor detection as a covariate. Mixed model ANCOVA was used to evaluate differences in means over time. Changes in FMBV over time within dietary groups were calculated using paired t-tests. Correlations were calculated using Spearman’s correlation coefficient (r_s_). Due to small sample sizes and a severe reduction of power by testing data for interaction terms^44^, the α level was adjusted to consider *p* < 0.15 as statistically significant for interaction effects. A *p*-value < 0.05 was considered statistically significant for all other analyses, and data are reported for both unadjusted and adjusted (for body weight at tumor detection) models as mean ± SEM unless otherwise stated.

## Results

### Tomato carotenoids were equivalent between CON and OB diets

Carotenoid profiles were similar between TP and TP-containing diets, and concentrations were similar between control (CON-TP) and obesogenic (OB-TP) diets (Supplementary Fig. S1).

### Histological outcomes

All tumors evaluated were grade 7 (poorly differentiated) according to the grading scheme used and previously described^45^. Approximately half the tumors retained an epithelial morphology, retaining rudimentary delineation of lobules and a densely packed epithelial morphology in which the cells had large, pleomorphic ovoid nuclei with “open” chromatin patterns. In other tumors, however, areas of neuroendocrinoid morphology were observed, in which the neoplastic cells were smaller and less pleomorphic, with better defined cell borders and smaller, rounder and more basophilic nuclei. A small percentage of tumors were entirely or almost entirely neuroendocrinoid in morphology.

### Dietary tomato led to paradoxical responses in CON vs. OB diets

**Figure 2:**
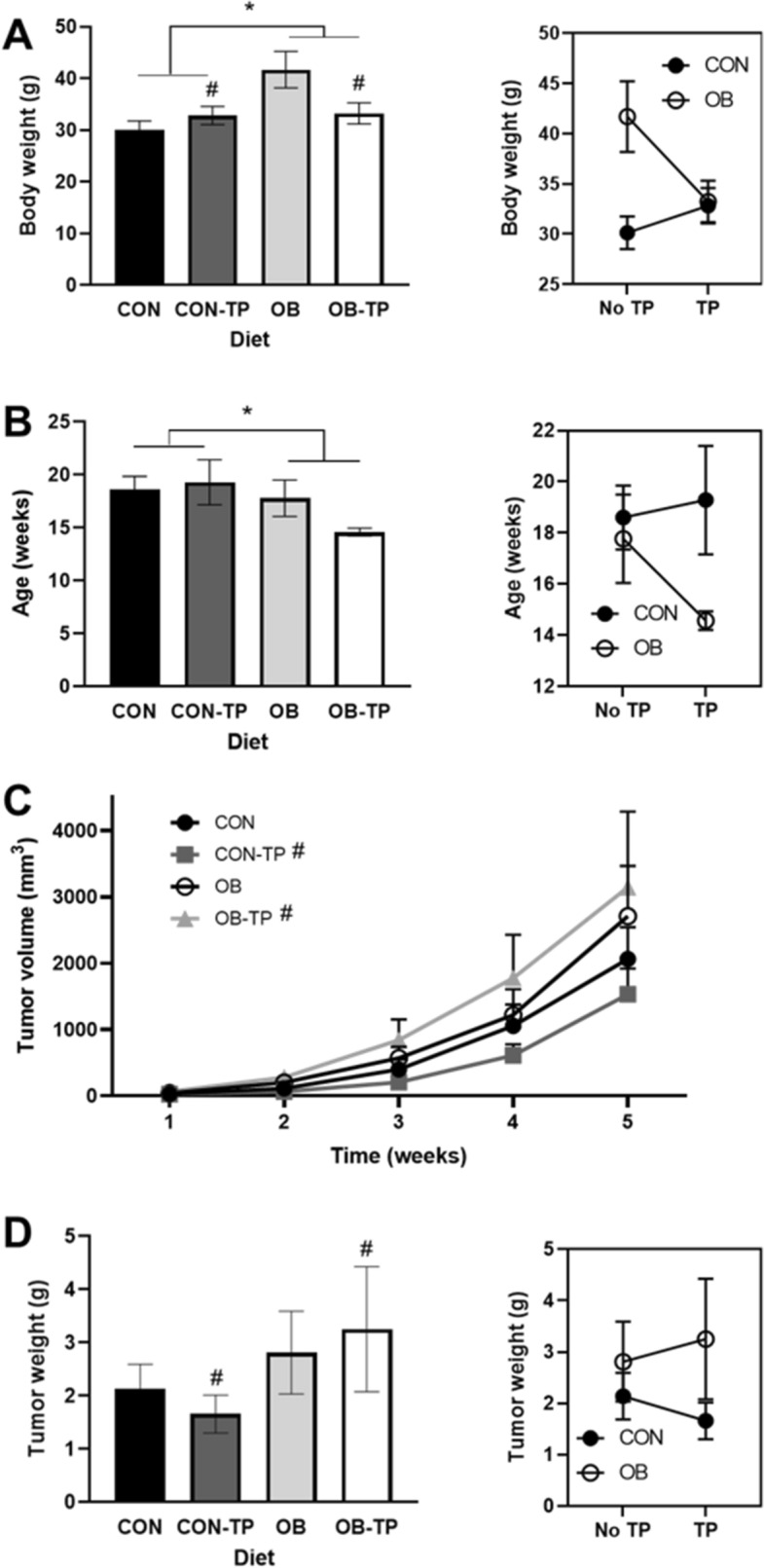
(**A**) Body weight and (**B**) age at tumor detection with corresponding interaction plots. A reversal of effect indicates that tomato powder (TP) led to divergent effects depending on incorporation with control (CON) or obesogenic (OB) diets. (**C**) Tumor volume (mm3) over time. Tumor growth rate was not significantly different between dietary groups after adjusting for body weight at tumor detection, but tumor volume at each time point was significantly affected by TP depending on diet. D) Tumor weight at euthanasia was differentially affected by TP depending on incorporation with CON or OB diets. *Indicates statistical significance (*p* < 0.05) and ^#^indicates a significant interaction effect (*p* < 0.15; by mixed-model ANCOVA with body weight at tumor detection as a covariate, n = 5/dietary group).

Regardless of TP inclusion, OB diets led to greater body weight gain with proportionate increases in periprostatic adipose (data not shown). OB-fed animals exhibited greater body weight at tumor detection (37.7 ± 2.4 g) compared with CON-fed animals (31.5 ± 1.2 g; *p* = 0.022; Fig. 2A). TP presence vs. absence within the OB and CON diets led to a significant interaction effect on body weight (*p* = 0.032). Specifically, within the OB diet groups, the presence of TP led to lower body weight at tumor detection. In contrast, within the CON diet groups, TP presence led to higher body weight at tumor detection.

Both greater body weight (*p* = 0.029) and OB diets led to tumor detection at an earlier age (16.2 ± 1.0 weeks) compared with animals fed CON diets (18.9 ± 1.2 weeks; *p* = 0.008; Fig. 2B). Body weight significantly affected tumor volume at detection, with greater body weight associated with greater tumor volume at detection and at each subsequent weekly tumor volume measurement (*p* = 0.001). After adjusting for body weight to investigate diet-dependent effects on tumor growth, OB and CON diets were not associated with tumor volume (Fig. 2C). Similarly, greater body weight tended to be associated with higher tumor weight at euthanasia (*p* = 0.053). After adjusting for body weight, diet did not significantly affect tumor weight (Fig. 2D). A significant interaction effect indicated that TP incorporation within the CON diet led to reduced tumor volume at detection, while TP incorporation into the OB diet led to greater tumor volume at detection (*p* = 0.004). These effects persisted across each weekly tumor volume measurement and at euthanasia. Complete results for both the unadjusted and adjusted models can be found in Supplementary Table 3.

### Dietary tomato reduced tumor blood perfusion over time as measured by UMI

**Figure 3:**
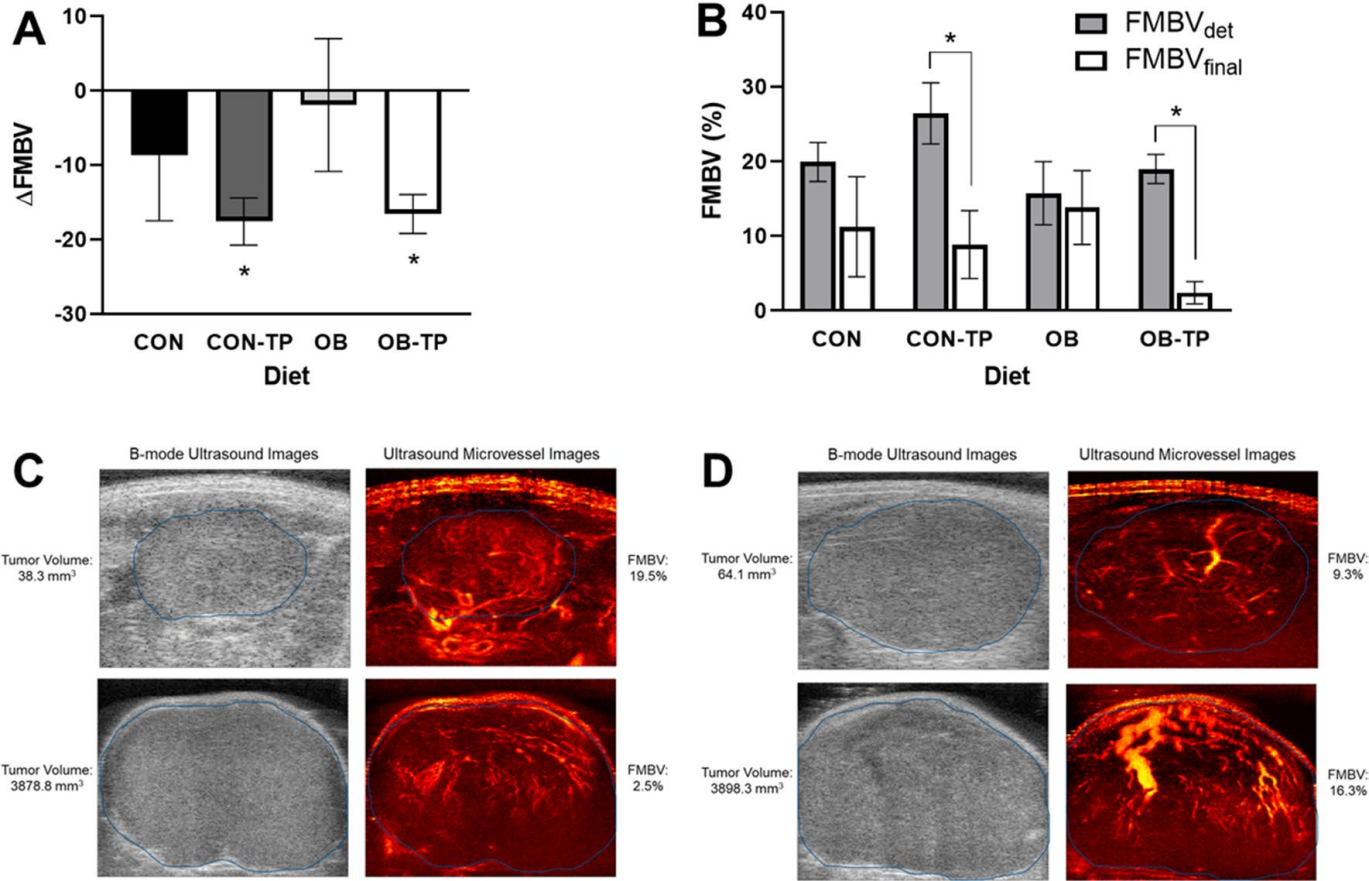
(**A**) Fractional moving blood volume (FMBV) overall changes (ΔFMBV) and (**B**) FMBV changes over time by dietary group show TP led to reduced tumor blood perfusion. Representative images depicting (**C**) decreasing FMBV and (**D**) increasing FMBV over time (between tumor detection and final tumor scan). Tumors are bordered in blue. *Indicates statistical significance (*p* < 0.05 by paired t-test FMBV, n = 5/dietary group).

Weekly tumor growth was monitored by ultrasound imaging, during which time IQ-data cineloops were captured for microvessel image processing. FMBV at tumor detection (FMBV_det_) and FMBV at week 5 of tumor growth (FMBV_final_) were not significantly different between dietary groups, but ΔFMBV was greater for animals fed TP-containing diets (*p* = 0.043; Fig. 3A). Specifically, a much larger decrease in tumor blood perfusion between FMBV_det_ and FMBV_final_ was observed for CON-TP (− 17.6 ± 3.2%) and OB-TP (− 16.6 ± 2.6%) groups when compared with CON (− 8.7 ± 8.8%) and OB (− 1.9 ± 8.9%) groups (Fig. 3B). Representative UMI images depicting decreasing and increasing changes in FMBV are shown in Fig. 3C,D. Tumor weight at euthanasia was negatively correlated with ΔFMBV (r_s_ = − 0.520, *p* = 0.019), showing that as tumors became larger, FMBV also decreased. This is consistent with tumor growth characteristics, as larger tumors become unable to sustain blood flow throughout the rapidly growing tumor^46^.

Interestingly, CON-TP animals developed the smallest tumors yet experienced the greatest reductions in tumor blood perfusion, and OB-TP animals developed the largest tumors while experiencing similar reductions in tumor blood perfusion. This suggests that TP inhibits angiogenesis in animals fed control and obesogenic diets, but that TP may promote tumor growth by alternative mechanisms when delivered within an obesogenic diet.

### Immunohistochemistry validated UMI sensitivity to detect microvascular blood flow

**Figure 4:**
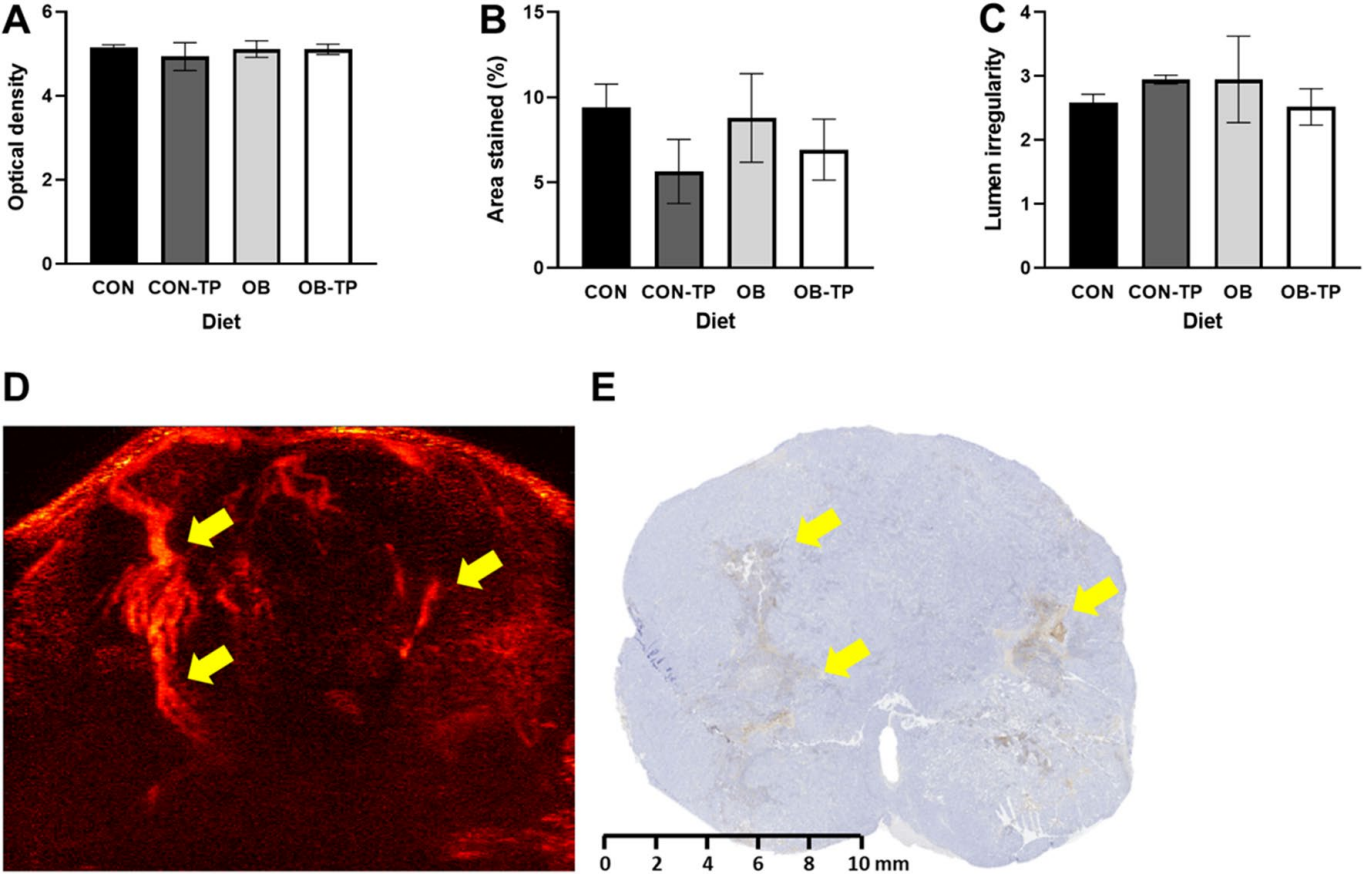
(**A**) Optical density and (**B**) density of intratumoral endothelial cell staining. Numeric trends indicate reduced microvessel density in TP-fed groups. (**C**) Lumen irregularity, calculated as: perimeter2/(4 × π × area), with a value of 1.0 indicating a perfect circle and values > 1.0 indicating increasing irregularity. (**D**) Ultrasound microvessel image showing blood flow through microvasculature. Yellow arrows indicate regional similarities observed by (**E**) immunohistochemistry staining against endothelial cells. *Indicates statistical significance (*p* < 0.05 by mixed-model ANCOVA with body weight at tumor detection as a covariate, n = 5/dietary group).

Neoangiogenic properties indicating increasing angiogenic potential include increased expression of endothelial cells (factor viii +), increased MVD, and greater lumen irregularity^23^. Factor viii expression did not differ between diets (Fig. 4A). TP inclusion in diets led to numerically reduced MVD, as indicated by reductions in total area of factor viii expression, compared to the respective CON and OB diets lacking TP (Fig. 4B). These observations are consistent with the reduction in FMBV observed by TP. Blood vessel lumen irregularity scores were not significantly different between diets (Fig. 4C) but were negatively correlated with ΔFMBV (r_s_ = − 0.447, *p* = 0.049), showing that as blood vessel lumen irregularity increased, FMBV decreased.

Observational examination of blood vessel staining against factor viii in a center cross-section of each tumor showed regional similarities in blood vasculature with those seen in UMI (Fig. 4D,E). This suggests UMI sensitivity to detect vessel-specific blood flow in an autochthonous and clinically relevant model of PCa.

### Dietary tomato reduced VEGF and markers of inflammation in animals fed CON diets

**Figure 5:**
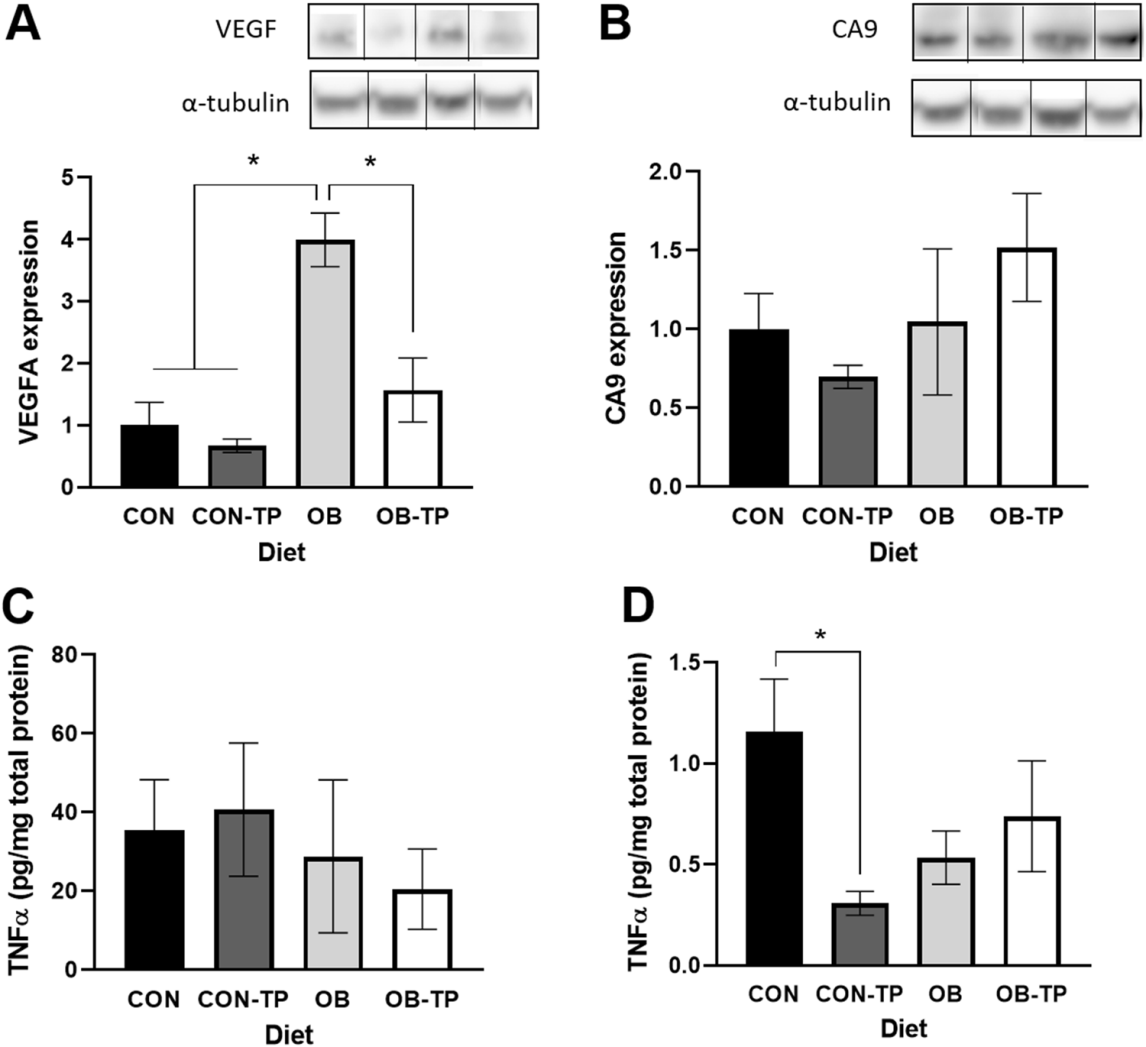
(**A**) Intratumoral VEGF and (**B**) CA9 expression relative to α-tubulin, with representative Western blot bands (CON, CON-TP, OB, OB-TP). Full-length, uncropped images are included within Supplementary Materials. OB diets significantly increased VEGF, and TP led to reduced VEGF, data which corroborates UMI findings of reduced tumor blood perfusion. (**C**) Periprostatic adipose expression of TNFα was variable and not associated with diet. (**D**) Intratumoral TNFα was reduced by TP only when incorporated into the CON diet. *Indicates statistical significance (*p* < 0.05 by mixed-model ANCOVA with body weight at tumor detection as a covariate, n = 5/dietary group).

The OB diet led to a significant increase in intratumoral VEGF expression (*p* = 0.001, Fig. 5A) compared with all other dietary groups. VEGF expression was comparatively reduced within tumors of animals fed TP-containing diets, which supports the UMI findings of reduced FMBV in tumors from TP-fed animals. While TP inclusion within the CON diet tended to reduce hypoxia-induced CA9 expression, the opposite effect was observed with TP incorporation into the OB diet (Fig. 5B).

Although periprostatic adipose TNFα was not significantly different between dietary groups and demonstrated no discernable dietary trends (Fig. 5C), intratumoral TNFα was significantly lower in animals fed the CON-TP diet than in animals fed the CON diet (*p* = 0.019) but was not significantly different between animals fed OB diets (Fig. 5D). However, a similar reversal of effect can be observed here, wherein TP was associated with numerically greater intratumoral TNFα in animals fed OB diets. Immunohistochemical staining of intratumoral infiltration of macrophages showed no significant differences between dietary groups, although numeric trends suggest TP may play a role in attenuating macrophage infiltration into tumor tissues (Supplementary Fig. S2).

### OB diets led to reduced expression of androgen-regulated genes

**Figure 6:**
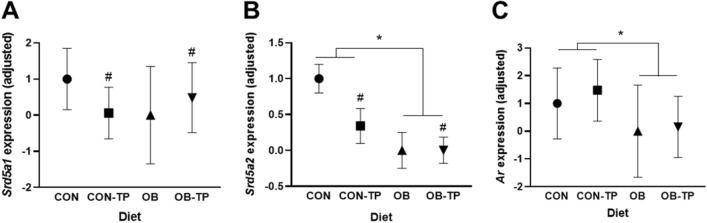
Changes in gene expression of (**A**) *srd5a1*, (**B**) *srd5a2*, and (**C**) *ar* by dietary group, after adjusting for body weight at tumor detection. *Indicates statistical significance (*p* < 0.05) and ^#^indicates a significant interaction effect (*p* < 0.15; by mixed-model ANCOVA with body weight at tumor detection as a covariate, n = 5/dietary group).

Although gene expression levels were lower with OB diets, higher body weight at tumor detection increased the expression of intratumoral *srd5a1* (*p* = 0.023) and *srd5a2* (*p* = 0.001). After adjusting for body weight at tumor detection, OB diets did not affect *srd5a1* expression (Fig. 6A) but led to significantly lower intratumoral *srd5a2* (*p* = 0.0004; Fig. 6B) and *ar* (*p* = 0.032, Fig. 6C) expression compared with CON diets. A significant interaction was observed whereby TP reduced both *srd5a1* (*p* = 0.030) and *srd5a2* (*p* = 0.016) expression in CON-fed animals and increased *srd5a1* and *srd5a2* expression in OB-fed animals.

## Discussion

This pilot study evaluated the effects of diet-induced obesity and tomato intake on advanced prostate tumor growth and longitudinal measurements of tumor blood perfusion; the latter measured using a novel application and modifications to our previously published high-frequency UMI technique^30^. We found that, although neither CON nor OB diets significantly affected tumor volumes, greater body weight was significantly associated with greater tumor size at each week of measurement. Interestingly, after adjusting for body weight at tumor detection, dietary tomato resulted in divergent effects when incorporated into CON or OB diets. TP significantly reduced tumor volume measurements in animals fed a CON diet, consistent with our previous findings assessing the impact of TP on prostate carcinogenesis in lean TRAMP animals^47,48^. TP, when incorporated into the CON diet, also led to significantly reduced tumor blood perfusion over time (as measured by UMI) and reduced intratumoral inflammation. The UMI-based measurements of tumor blood perfusion were validated by histological gold-standard measurements of factor viii and blood vessel lumen irregularity scores. Furthermore, TP-containing diets led to reduced expression of intratumoral VEGF, supporting the reduction in FMBV observed by UMI and further corroborating UMI sensitivity to detect morphological and functional changes of the vascular architecture. These effects by dietary tomato are promising, as PCa metastasis and mortality have been found to be strongly correlated with tumor angiogenesis^49^ and inflammation^50^.

Consistent with effects of TP in the CON diet, TP incorporation into the OB diet also led to reduced tumor blood perfusion and a significant reduction in the expression of VEGF. However, these results were observed congruently with increased tumor volume compared to animals fed the OB diet excluding TP. Molecular trends examining hypoxia-induced CA9 expression suggest that TP inclusion within the OB diet tended to increase intratumoral hypoxia compared with tumors from animals fed the OB diet. These results suggest that, while TP inhibits angiogenesis, this effect appears to be beneficial only in the context of a control diet and potentially deleterious when TRAMP mice are fed an obesogenic, Western-style diet. The role of dietary influence in mediating tumor responses to anti-angiogenic therapies may help to partially explain the mixed results observed by inhibiting angiogenesis in PCa^17–22^.

Although OB diets led to significantly greater body weight at tumor detection, OB-TP body weights were only marginally greater than CON-TP body weights. These data are consistent with observations showing inhibition of adipogenesis and promotion of fat oxidation by carotenoids^51,52^. As such, the interaction of TP with the altered nutritional composition of the OB diet did not lead to enhanced body weight but was associated with greater tumor size. Western-style diets, such as the OB diet used in this study, modify metabolic regulation and induce an inflammatory phenotype^53^. Carotenoids found in tomatoes, such as lycopene, may act as antioxidants to neutralize the reactive oxygen species produced by inflammatory responses. However, in highly inflammatory environments, carotenoid oxidation products may have cytotoxic effects^54^. Lycopene cleavage products have only been associated with chemoprotective effects to date^55^, but the anti-angiogenic effects of TP in a growth-promoting tumor microenvironment may lead to enhanced intratumoral hypoxia, as evidenced by reduced tumor blood perfusion and greater CA9 expression, and subsequent downstream signaling to promote tumor growth.

Interestingly, we did not observe increased inflammation either with OB diets or with increased body weight. However, interesting visual trends in macrophage infiltration into tumor tissues were observed by immunohistochemical staining, suggesting that OB diets lead to greater tumor infiltration of inflammatory cells. While TNFα is a good marker for inflammation in adipose tissue^56^, other inflammatory markers, such as IL-6, have been shown to be elevated in patients with PCa^57^. Additionally, adipose tissue secretes other pro-inflammatory cytokines, such as IL-1 and leptin^56^, that have been found to play a role in aggravating cancer growth. Current work involves further exploration into the inflammatory changes mediated by obesogenic diets and increased adiposity as well as the potential ameliorating roles by TP.

Consistent with previously reported results^58^, we observed that increased body weight was positively associated with gene expression of the steroid-regulated genes *srd5a1* and *srd5a2*. Despite this positive association, intratumoral androgen-regulated gene expression levels remained drastically reduced in animals fed OB diets. Because obesity^59^ and high-fat diets^60^ are associated with low androgens, the subsequent reduced activation of the AR may lead to its downregulated expression in prostatic tissues^61^. Indeed, we observed reduced *ar* expression with OB diets. Reduced activity of the AR can then result in the reduced expression of *srd5a1* and *srd5a2*, which encode the 5α-reductase enzymes (isoform 1 and isoform 2) responsible for catalyzing the conversion of androgens to their more potent forms. These data may partially explain the low efficacy of 5α-reductase inhibitors in men with obesity^62^.

*Srd5a2* is the predominant isoform in the normal prostate; while the underlying mechanistic advantage is unclear, *srd5a2* expression is downregulated in PCa while *srd5a1* expression is upregulated^63^. We have previously shown that TP reduced serum testosterone in rodent models^64,65^ as well as reduced prostatic expression of both *srd5a1* and *srd5a2*^66^. In this study, we also observed a significant reduction of intratumoral *srd5a1* and *srd5a2* by TP between mice fed CON diets, indicating that protection against PCa by dietary tomato is mediated, in part, by TP disruption of the androgen axis. However, the opposite effect was seen with TP incorporation into the OB diet, wherein *srd5a1* and *srd5a2* expression were increased with TP intake. Taken together, these results suggest that TP disrupts androgen metabolism when included within control diets yet enhances androgen metabolism when included within obesogenic diets.

In this work, we report that UMI can be used to non-invasively assess microvascular tumor perfusion in vivo and can therefore potentially evaluate the effects of anti-angiogenic and vascular targeting agents on PCa tumors at all stages of therapy. While anti-angiogenic treatments such as tyrosine kinase inhibitors (TKI) are effective for some cancers^67^, studies have shown resistance to anti-angiogenic TKIs in some prostate tumors^68,69^. Furthermore, prostate tumors are notoriously heterogeneous, with a current lack validated clinical biomarkers to select PCa patients for either ADT or anti-angiogenic therapy^70,71^. Response to treatment is instead measured by tumor growth metrics, data which can be collected simultaneously with IQ-data to assess markers of angiogenesis and to help guide individualized treatment course. Although the implementation of UMI presented in this work was restricted to line-by-line acquisitions with a pre-clinical ultrasound system, the recent emergence of ultrafast plane-wave imaging in the clinical setting significantly increases the sensitivity to microvascular flow and provides improved tissue rejection without the need for invasive contrast agent injections. As such, UMI can potentially serve as an inexpensive, safe, and rapid screening tool for anti-angiogenic therapy selection for PCa patients.

This study does present with some limitations. First, the selection of TRAMP animals as a model for prostate adenocarcinoma development has been previously challenged^72^. TRAMP animals have been observed to develop prostate tumors with NE features, whereas NE tumors make up only a small percentage of primary prostate tumors. However, the NE phenotype has been shown to develop as PCa tumors progress and has been shown to retain features associated with adenocarcinoma, as shown in this study, and represents an advanced PCa lesion similar to those observed in human PCa^73^. Next, although we were able to detect significant changes in vascular perfusion with the UMI technique, the small sample size used in this pilot study was not sufficiently statistically powered to detect minute changes in molecular outcomes. However, despite the small sample size, we did observe significant molecular effects not only for intratumoral TNFα and VEGF expression, but also for *srd5a1*, *srd5a2*, and *ar* expression. These results imply that there is a substantial interaction between diet and angiogenesis, inflammation, and the androgen axis. This study also resulted in interesting trends between diet, body weight, and intratumoral hypoxia and inflammation which can serve as hypothesis-generating observations to be explored in ongoing and future work involving larger sample sizes. Current work involves an assessment of the lipid profiles between dietary groups and an in-depth exploration into the association between periprostatic adipose tissue inflammation and tumor growth. Additionally, while we were able to visually assess similar regions of UMI-detected vasculature with gold-standard immunohistochemistry (Fig. 4), it was impossible to truly co-register the two vasculature detection techniques due to the handling and processing of tissue sections for histology. The different sampling volumes of the relatively thick ultrasound imaging plane in comparison to a thin histological section resulted in a general overestimation of FMBV versus IHC MVD. Furthermore, there may be an imperfect correlation between histological microvascular density and functional vasculature, such that endothelial cell ‘hot-spots’ in the tumor do not necessarily indicate increased blood perfusion. Future studies will involve more precise and controlled tissue fixation handling to maintain the ultrasound imaging cross-section, potentially with fiducial and/or 3D surface-based registration^74^ of histology with ultrasound. Future studies may also involve super-resolution UMI to further enhance microvessel visibility and quantitatively measure blood flow speed in vivo. These measurements may further assist with characterizing tumor blood flow and the associated tumor microenvironment.

This pilot study demonstrated the feasibility of using the UMI technique for the non-invasive measurement of tumor blood perfusion. The results reported in this study also support existing pre-clinical data indicating that dietary tomato is an effective anti-angiogenic agent^23,25–27^, leading to overall reduced tumor blood perfusion over time. However, these anti-angiogenic effects may paradoxically affect tumor growth, leading to enhanced tumor growth in the context of obesity and/or Western-style diets. While dietary tomato has been found to be protective against developing PCa, some studies have shown this protection to be limited in Western populations^75^. The results presented here warrant further investigation and suggest that the protective effects elicited by TP (inhibition of angiogenesis, reduced intratumoral inflammation, and reduced tumor growth) disappear when incorporated with a Western-style diet. In fact, when combined with a Western-style diet, TP may be associated with enhanced deleterious features indicative of more advanced PCa such as increased androgen metabolism as well as reduced angiogenesis with associated enhanced tumor growth.

## Supplementary Information

Supplementary Information.
