# Supplementary material for: Dietary tomato inhibits angiogenesis in TRAMP prostate cancer but is not protective with a Western-style diet in this pilot study
Source: Sci Rep. 2021 Sep 17;11:18548. doi: 10.1038/s41598-021-97539-2 (PMC8448771; doi:10.1038/s41598-021-97539-2)
Supplement: Supplementary file 1 — Supplementary Information. [file 41598_2021_97539_MOESM1_ESM.docx]

**Dietary tomato inhibits angiogenesis in TRAMP prostate cancer but is not protective with a Western-style diet in this pilot study**

Catherine C. Applegate^1^*, Matthew R. Lowerison^2,3^, Emma Hambley^1†^, Pengfei Song^2,3,4^, Matthew A. Wallig^1,5^, John W. Erdman Jr.^1,2,4,5^*

^1^Division of Nutritional Sciences, University of Illinois at Urbana-Champaign, Urbana, IL 61801, USA.

^2^Beckman Institute for Advanced Science and Technology, University of Illinois at Urbana-Champaign, Urbana, IL 61801, USA.

^3^Department of Electrical and Computer Engineering, University of Illinois at Urbana-Champaign, Urbana, IL 61801, USA.

^4^Cancer Center at Illinois, University of Illinois at Urbana-Champaign, Urbana, IL 61801, USA.

^5^Department of Pathobiology, College of Veterinary Medicine, University of Illinois at Urbana−Champaign, Urbana, IL 61801, USA*.*

^6^Department of Food Science and Human Nutrition, University of Illinois at Urbana-Champaign, Urbana, IL 61801, USA.

**^†^Current affiliation:** Division of Biology, Kansas State University, Manhattan, KS 66506, USA.

***Corresponding authors:** [cca2@illinois.edu](mailto:cca2@illinois.edu), [jwerdman@illinois.edu](mailto:jwerdman@illinois.edu)

**Supplementary Table S1.** Experimental diet composition. Diets are powdered and modified to ensure consistent macronutrient composition between non-TP and TP diets.

| Ingredient | AIN-93G (%w/w) | | Obesogenic (%w/w) | |
| --- | --- | --- | --- | --- |
|  | **No TP** | **10% TP** | **No TP** | **10% TP** |
| Cornstarch | 39.0% | 36.3% | 5.6% | 3.3% |
| Casein | 19.6% | 17.7% | 19.2% | 17.2% |
| Maltodextrin | 13.0% | 10.5% | 5.9% | 3.6% |
| Sucrose | 9.8% | 9.7% | 33.4% | 33.0% |
| Cellulose | 4.9% | 4.1% | 4.9% | 4.1% |
| Mineral mix | 3.4% | 3.4% | 4.2% | 4.2% |
| Vitamin mix | 1.0% | 1.0% | 1.9% | 1.8% |
| L-cystine | 0.29% | 0.29% | 0.29% | 0.29% |
| Choline bitartrate | 0.25% | 0.24% | 0.29% | 0.29% |
| Soybean oil | 7.0% | 6.8% | 2.0% | 1.9% |
| Anhydrous milkfat | 0.0% | 0.0% | 20.6% | 20.4% |
| Tomato powder | 0.0% | 10.0% | 0.0% | 10.0% |
| Water | 1.8% | 0.0% | 1.8% | 0.0% |
| **kcal/g** | **3.89** | **3.85** | **4.61** | **4.55** |

*By proximate analysis, 10.0 g TP provides 4.5 g total carbohydrates, 1.7 g crude protein, 0.1 g crude fat, 0.8 g crude fiber, 1.8 g water, and 1.1 g ash.

**Supplementary Table S2.** PCR primer sequences.

| **Gene** | **Forward Primer (5’🡪3’)** | **Reverse Primer (3’🡪5’)** |
| --- | --- | --- |
| *pbsn* | CCG GTC GAC CGG AAG CTT CCA CAA GTG CAT TTA |  |
| *sv40tag* | CTC CTT TCA AGA CCT AGA AGG TCC A |  |
| *csn2* | GAT GTG CTC CAG GCT AAA GTT | AGA AAC GGA ATG TTG TGG AGT |
| *ar* | CTG GGA AGG GTC TAC CCA C | GGT GCT ATG TTA GCG GCC TC |
| *srd5a1* | GAG TTG GAT GAG TTG CGC CTA | GGA CCA CTG CGA GGA GTA G |
| *srd5a2* | GAT CCT GTG CTT TGG GAA ACC | GCA TCC CTA CCG ACA CCA C |


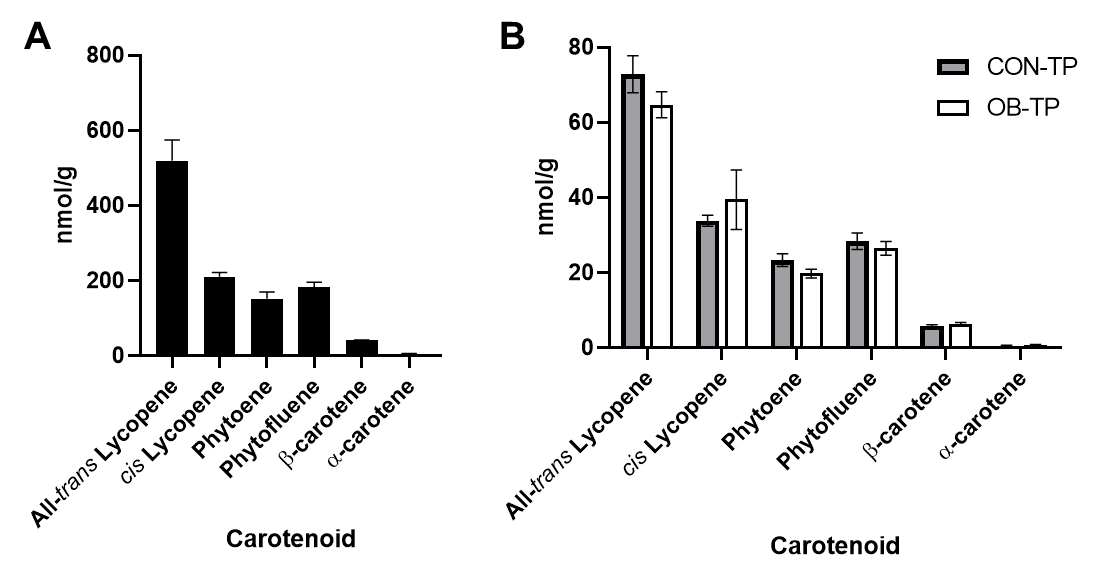


**Supplementary Figure S1.** Carotenoid content in (A) tomato paste (TP); and (B) control (CON) and obesogenic (OB) diets. Carotenoid concentrations did not differ between diets by Student’s t-test (n=3).


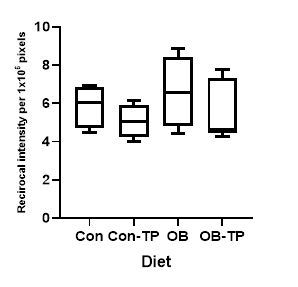


**Supplementary Figure S2.** Tumor macrophage infiltration (anti-F4/80) by dietary group. Data are shown as mean of the reciprocal intensity normalized to the number of pixels ± SEM. Data are not statistically significant (*p*<0.05 by one-way ANOVA, n=5/diet).

| **Outcome** | **CON** | |  | **OB** | |  | ***p*-values^2^** | | | |
| --- | --- | --- | --- | --- | --- | --- | --- | --- | --- | --- |
|  | No TP | TP |  | No TP | TP |  | Diet | TP | Diet*TP | Body weight^3^ |
| Body weight (g)^3^ | 30.1±1.6 | 32.8±1.8 |  | 41.7±3.5 | 33.2±2.1 |  | **0.022** | 0.240 | **0.032** |  |
| Age (weeks)^3^ | 18.6±1.3 | 19.3±2.1 |  | 17.8±1.7 | 14.6±0.4 |  | 0.086 | 0.419 | 0.218 |  |
| *Adjusted* | 20.1±1.5 | 19.8±1.3 |  | 15.3±1.7 | 15.0±1.3 |  | **0.008** | 0.842 | 0.967 | **0.029** |
| Tumor volume (mm^3^)^3^ | 26.8±7.6 | 24.3±6.6 |  | 42.1±13.2 | 65.7±18.0 |  | **0.034** | 0.401 | 0.300 |  |
| *Adjusted* | 43.3±9.4 | 30.6±8.7 |  | 14.6±10.8 | 70.4±8.6 |  | 0.591 | **0.029** | **0.004** | **0.001** |
| Tumor weight (g) | 2.14±0.45 | 1.66±0.36 |  | 2.81±0.78 | 3.25±1.18 |  | 0.156 | 0.980 | 0.555 |  |
| *Adjusted* | 2.80±0.76 | 1.91±0.70 |  | 1.71±0.87 | 3.44±0.70 |  | 0.795 | 0.568 | **0.123** | 0.053 |

**Supplementary Table S3.** Summary of pre-clinical results^1^

^1^Results are shown as unadjusted and adjusted (using body weight as a covariate) values for level of diet (CON or OB), level of TP (with or without), and the interaction between diet and TP (diet*TP).

^2^Significant values in bold as measured by mixed-model ANCOVA using body weight at tumor detection as a covariate (n=5/dietary group).

^3^At tumor detection

Abbreviations: CON: control; OB: obesogenic; TP: tomato powder

**Original Western blot images:**

VEGFA:


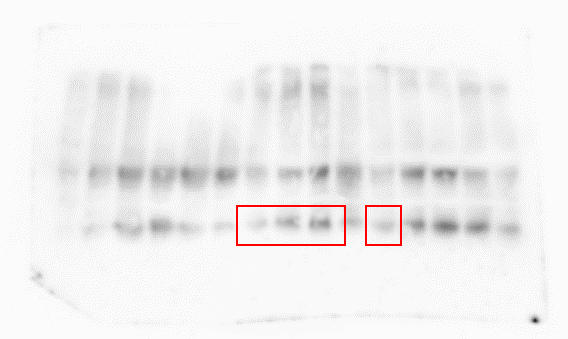


CA9:


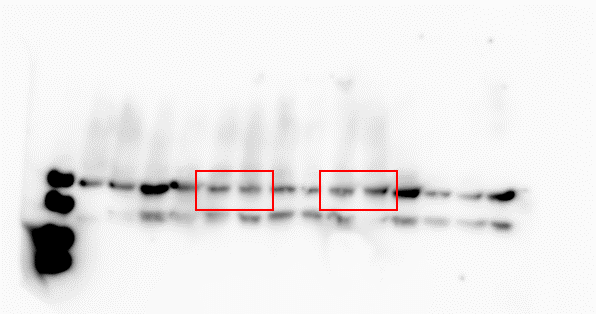


α-tubulin (loading control):


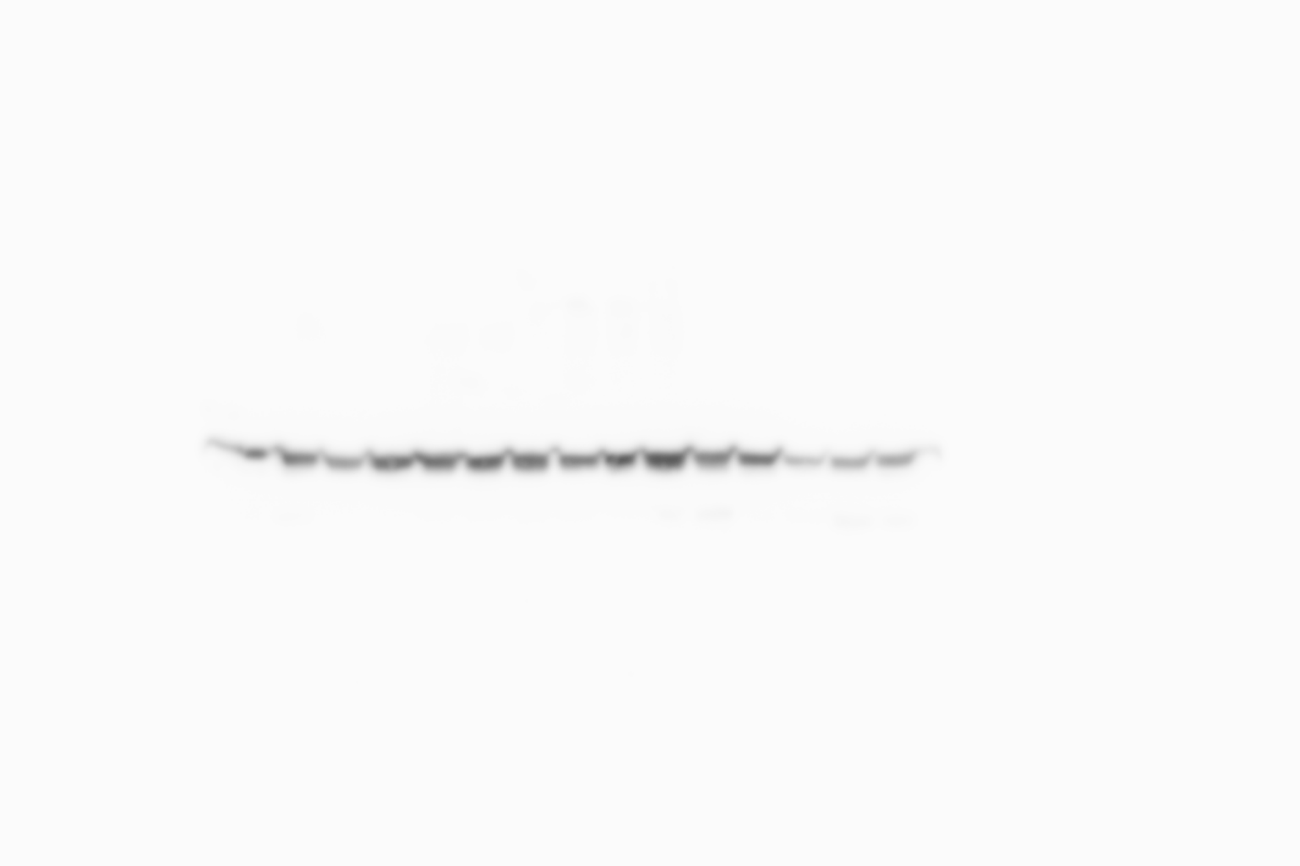


| **ID** | **Diet** | **Tumor volume at euthanasia (mm^3^)** | **Tumor weight at euthanasia (g)** | **Animal weight at euthanasia (g)** |
| --- | --- | --- | --- | --- |
| **63** | CON | 1413.995 | 1.4094 | 29 |
| **84** | CON | 2214.341 | 1.5587 | 29 |
| **67** | CON | 3878.765 | 2.1321 | 37 |
| **79** | CON | 1370.722 | 3.8918 | 33 |
| **181** | CON | 1447.093 | 1.6984 | 33.4 |
| **6** | CON-TP | 2731.84 | 2.7283 | 34 |
| **21** | CON-TP | 772.631 | 0.8768 | 34 |
| **72** | CON-TP | 1910.339 | 2.0474 | 36 |
| **138** | CON-TP | 1636.775 | 1.771 | 28 |
| **147** | CON-TP | 601.661 | 0.874 | 35 |
| **119** | OB | 712.788 | 0.7339 | 32 |
| **97** | OB | 2520.563 | 4.6235 | 48 |
| **82** | OB | 4615.911 | 1.2565 | 40 |
| **154** | OB | 3112.748 | 3.2743 | 49 |
| **165** | OB | 3898.348 | 4.171 | 53.3 |
| **41** | OB-TP | 3778.423 | 4.1175 | 43 |
| **85** | OB-TP | 285.937 | 0.2654 | 22 |
| **104** | OB-TP | 7036.235 | 7.1786 | 35 |
| **112** | OB-TP | 2980.905 | 3.0211 | 33 |
| **123** | OB-TP | 1648.784 | 1.6712 | 35 |

**Supplementary Table S4.** Animal ethics metrics.
